# Exploring productivity and collaboration in Australian Indigenous health research, 1995–2008

**DOI:** 10.1186/1478-4505-11-42

**Published:** 2013-11-08

**Authors:** Alice R Rumbold, Joan Cunningham, Brydie Purbrick, Jenny M Lewis

**Affiliations:** 1 Robinson Institute, The University of Adelaide, Adelaide, SA 5005, Australia; 2 Epidemiology and Health Systems Division, Menzies School of Health Research, Charles Darwin University, Casuarina, PO Box 41096, Darwin, NT 0811, Australia; 3 School of Social and Political Sciences, The University of Melbourne, Parkville, Melbourne, VIC 3010, Australia

**Keywords:** Aborigines, Australian, Cooperative behaviour, Research personnel

## Abstract

**Background:**

Building research capacity in Indigenous health has been recognised as integral in efforts to reduce the significant health disparities between Indigenous and other Australian populations. The past few decades have seen substantial changes in funding policy for Australian Indigenous health research, including increases in overall expenditure and a greater focus on collaborative and priority-driven research. However, whether these policy shifts have resulted in any change to the structure of the research workforce in this field is unclear. We examine research publications in Australian Indigenous health from 1995–2008 to explore trends in publication output, key themes investigated, and research collaborations.

**Methods:**

A comprehensive literature search was undertaken to identify research publications about Australian Indigenous health from 1995–2008. Abstracts of all publications identified were reviewed by two investigators for relevance. Eligible publications were classified according to key themes. Social network analyses of co-authorship patterns were used to examine collaboration in the periods 1995–1999, 2000–2004 and 2005–2008.

**Results:**

Nine hundred and fifty three publications were identified. Over time, the number of publications per year increased, particularly after 2005, and there was a substantial increase in assessment of health service-related issues. Network analyses revealed a highly collaborative core group of authors responsible for the majority of outputs, in addition to a series of smaller separate groups. In the first two periods there was a small increase in the overall network size (from n = 583 to n = 642 authors) due to growth in collaborations around the core. In the last period, the network size increased considerably (n = 1,083), largely due to an increase in the number and size of separate groups. The general size of collaborations also increased in this period.

**Conclusions:**

In the past few decades there has been substantial development of the research workforce in Indigenous health, characterised by an increase in authors and outputs, a greater focus on some identified priority areas and sustained growth in collaborations. This has occurred in conjunction with significant changes to funding policy for Indigenous health research, suggesting that both productivity and collaboration may be sensitive to reform, including the provision of dedicated funding.

## Background

Research aimed at improving the health and wellbeing of Aboriginal and Torres Strait Islander (Indigenous) peoples has been identified as a major priority in Australia [1]. Although there is a long history of Indigenous communities being the subject of research, past practices have often been exploitative, disrespectful and of little value to communities [2,3]. There has also been limited involvement of Indigenous people in the design and control of research. Only in the past few decades has there been a concerted effort to reform practices, with the development of ethical guidelines [4,5], a focus on collaborative research models and acknowledgement of the need to train more Indigenous researchers [3,6-8].

In conjunction with a reform agenda that supports greater Indigenous leadership of research, there has been a substantial change to funding policy in this area by Australia’s National Health and Medical Research Council (NHMRC). Since 1997, these changes have aimed not only to increase overall support for Indigenous health research but also to encourage a strategic and priority-driven approach. Increasing representation of Indigenous peoples across NHMRC committees, a commitment to expend at least 5 % of the annual budget on Indigenous health, and development and endorsement of “*The NHMRC Road Map: A Strategic Framework for Improving Aboriginal and Torres Strait Islander Health through Research”*[9] are some of the key strategies undertaken to achieve change [10].

These important policy developments have underscored the need to build research capacity in Indigenous health, which includes developing the research workforce (both Indigenous and non-Indigenous researchers). However, it is unclear whether they have resulted in any substantial change to the structure and dynamics of the workforce. Here, we explore the development of Indigenous health research in Australia during this period of policy change, by analysing research publications in Indigenous health between 1995 and 2008. We describe trends in publication output and major research themes investigated, as well as key journals publishing research articles. We also describe trends in research collaborations over this period by undertaking a network analysis of co-authorship patterns.

## Methods

### Identification of relevant publications

We undertook a literature search to identify publications about Australian Indigenous health research using the following online databases: PubMed; Aboriginal and Torres Strait Islander Health Bibliography; Australian Public Affairs Information Service – Aboriginal and Torres Strait Islander Subset; and Australian Medical Index. The search strategy employed and the methods used to identify relevant papers has been published previously [11,12]. Initially, we searched for publications in the years 1995 to 2004; this was subsequently expanded to include the years 2005 to 2008. Abstracts of all publications identified were reviewed by two investigators and papers were excluded if the focus was not Australian Indigenous health or not human health, or if the methodology indicated it was not an original research report.

### Classification of publications according to research themes

Eligible publications were classified according to key research themes. The classification was based on the title of the publication, and where it was unclear from the title, the abstract and/or full paper was reviewed. Publications were classified according to whether there was a focus on child health (yes/no), and a series of categories developed to describe the main exposure and the main outcome of interest. The categories of exposures included: i) substance use (e.g., alcohol, tobacco, petrol sniffing, other); ii) nutrition, obesity, physical activity, fetal/child growth and health promotion; iii) physical environment (e.g., housing, air, water); iv) social environment (e.g., racism, stress, grief, psychosocial, socioeconomic circumstances, prison); v) immunisation and screening; vi) health knowledge, attitudes, health literacy, patient cultural factors, individual care practices, experiences of care; vii) service-related issues (e.g., quality of care, data quality, management, needs assessment, staff attitudes, service culture, access, funding, evaluations, health workforce, research methodology); viii) medication (e.g., treatment trials); and ix) protective equipment. The categories for outcomes included: i) arthritis and musculo-skeletal conditions; ii) asthma; iii) cancer; iv) cardiovascular disease (including rheumatic fever), diabetes, renal disease, obesity and nutritional status; v) injury; vi) mental health; vii) ear disease; viii) oral health; ix) communicable diseases (e.g., sexually transmissible infections, pneumonia/influenza, skin disease); x) maternal and child health (e.g., birth outcomes, breastfeeding, child growth, perinatal and infant mortality); xi) general mortality (all causes, multiple causes), hospitalization (all causes, multiple causes) and general service use (including aged care); and xii) other diseases and conditions (including lung function, atopy and family functioning). Where multiple exposures or outcomes were assessed or it was unclear what the main exposure or outcome was, the publication was classified as having no main exposure or outcome.

Two authors initially independently classified 50 different publications each, and the above categories were reviewed for relevance and revised as necessary. The remaining publications were then divided between the two authors and classified. One author (AR) checked the classification of all publications for consistency between authors. Discrepancies were resolved through discussion and consensus.

### Network analyses

Social network analyses of co-authorship patterns among eligible publications were used to examine trends in research collaborations in the time period. Network approaches explore relational data and have been used to describe patterns of co-authorship and collaboration in a range of fields, including health services research [13,14].

Network maps were generated for the years 1995 to 1999, 2000 to 2004, and 2005 to 2008, based on the number of author pairings in each time period. Network maps consist of nodes which represent unique authors, and links, which represent co-authorship relationships (i.e., links from authors to authors). The thickness of the lines between the nodes (authors) represents the number of ties between authors such that a thicker line indicates a stronger relationship. The authors with the most links are placed at the centre of the map. Authors with no links (i.e., one or more single-author publications in the time period) are placed in the upper left hand corner.

Measures of components and density for each network map were also generated. Components are maximally connected sub-graphs, meaning that there are paths between all the nodes (which are authors in this case) [15]. Components are the parts of a network that are disconnected. If all authors have paths between them in a network, the network has only a single component, so the number of components is a measure of how many regions a network contains. The density of a network is a measure of its cohesion. It is the number of actual ties present (connections between nodes) compared with the total possible number in any network, if every node is directly connected to every other node [16]. A fully connected network would have a density of 1.

In order to develop network maps, each eligible publication was assigned a reference ID, and each unique author was assigned an author ID number. An Excel file was then developed to document the number of pairings between unique authors in a given year. Network maps and component and density measures were generated using the program Ucinet [16].

### Ethics approval

This study was approved by the Human Research Ethics Committee of the Menzies School of Health Research and the Northern Territory Department of Health, and its Aboriginal subcommittee.

## Results

**Figure 1:**
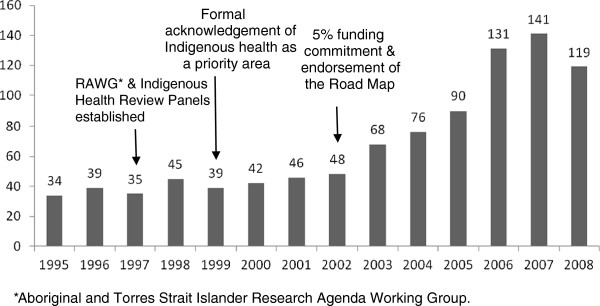
**Number of eligible publications per year in the study period, 1995 to 2008, and timing of key policy decisions by the National Health and Medical Research Council **[10]**.**

We identified a total of 953 eligible publications. Figure 1 shows the number of eligible publications per year, as well as the years in which key NHMRC policy decisions about Indigenous health were made. There was a steady increase in the number of eligible publications with increasing year; approximately two thirds were published from 2003 onwards. The majority (98%) were journal articles; the remaining publications were research monographs and reports. There were 256 unique sources of publications (n = 233 journals, 91%) contributing an average of 3.7 publications (standard deviation 11.4, range 1–121). The journals contributing the majority of articles to the network are listed in Table 1. The two journals contributing the most articles to the network were the *Medical Journal of Australia* and the *Australian and New Zealand Journal of Public Health*, with over a quarter of all publications occurring in these two journals combined.

**Table 1:** Journals in which eligible articles were published

**Journal title**	**n = 953**	**%**
Medical Journal of Australia	121	12.7
Australian and New Zealand Journal of Public Health	120	12.6
Journal of Paediatric and Child Health	39	4.1
Australian Journal of Rural Health	38	4.0
Aboriginal and Islander Health Worker Journal	34	3.6
Rural and Remote Health	19	2.0
Drug and Alcohol Review	17	1.8
Australian Journal of Primary Health	16	1.7
Clinical and Experimental Ophthalmology	15	1.6
Communicable Diseases Intelligence	15	1.6
Contemporary Nurse	12	1.3
Health Promotion Journal of Australia	12	1.3
Australian Family Physician	11	1.2
Australian Health Review	11	1.2
Pediatric Infectious Diseases Journal	11	1.2
Diabetes Research and Clinical Practice	10	1.0
Epidemiology and Infection	10	1.0
Kidney International	9	0.9
Australian and New Zealand Journal of Obstetrics and Gynaecology	8	0.8
Paediatric and Perinatal Epidemiology	8	0.8
Social Science and Medicine	8	0.8
Internal Medicine Journal	7	0.7
Australian and New Zealand Journal of Surgery	6	0.6
Australian and New Zealand Journal of Medicine	6	0.6
Diabetes Care	6	0.6
International Journal of Epidemiology	6	0.6
Nephrology	6	0.6
Vaccine	6	0.6
Australasian Psychiatry	5	0.5
BMC Health Services Research	5	0.5
Ethnicity and Disease	5	0.5
Heart Lung Circulation	5	0.5
Journal of Clinical Microbiology	5	0.5
Sexual Health	5	0.5
Other*	336	35.3

*Includes journals that had published fewer than 5 eligible articles in the time frame and publications that were monographs and reports.

Across the eligible publications there were 1,803 unique authors; most authors (65%) had only one eligible publication in the time period.

### Research themes

**Figure 2:**
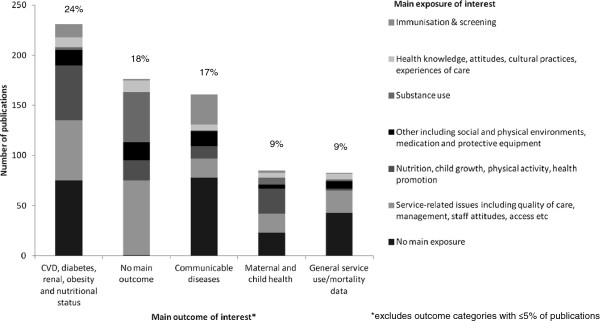
Classification of publications by main exposure and/or outcome.

Of the 953 publications, 226 (24%) had a specific focus on Indigenous child health. Figure 2 presents the classification of eligible publications by main outcome and main exposure of interest. In close to a quarter of all publications (24%), the main outcome was cardiovascular disease, diabetes, renal disease and/or obesity and nutritional status. Communicable diseases, maternal and child health and general service use/mortality data were also commonly assessed (17%, 9% and 9%, respectively). Mental health, cancer, ear disease, injury, oral health, asthma, arthritis and musculo-skeletal conditions, and other diseases and conditions were the least frequently assessed, with five percent or less of publications classified as having these outcomes as the main focus (data not shown in Figure 2).

Overall, the most common category of exposure was service-related issues (25%), followed by nutrition, child growth, physical activity and health promotion (13%), substance use (8%), health knowledge, attitudes, cultural practices and experiences of care (6%), immunisation and screening (6%), social environment (5%), physical environment (2%), medication (2%) and protective equipment (<1%). The proportion of each category of exposure varied across each outcome category (Figure 2).

There were 176 (18%) publications classified as having no main outcome of interest; among these publications, the most common exposures assessed were service-related issues (42%) or substance abuse (28%). There were 315 (33%) publications classified as having no main exposure of interest.

**Figure 3:**
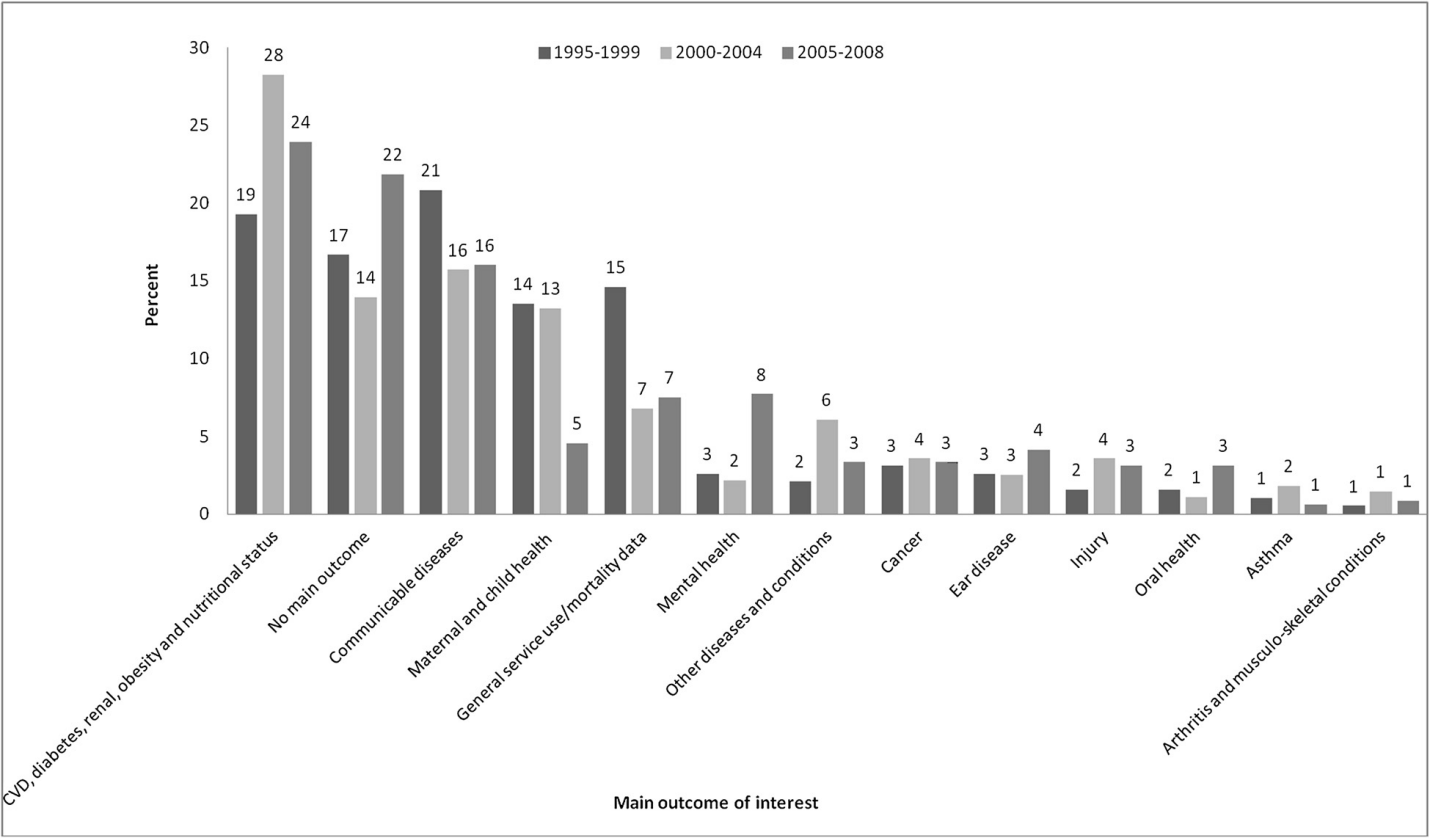
Proportion of publications in each outcome category across each time period.

**Figure 4:**
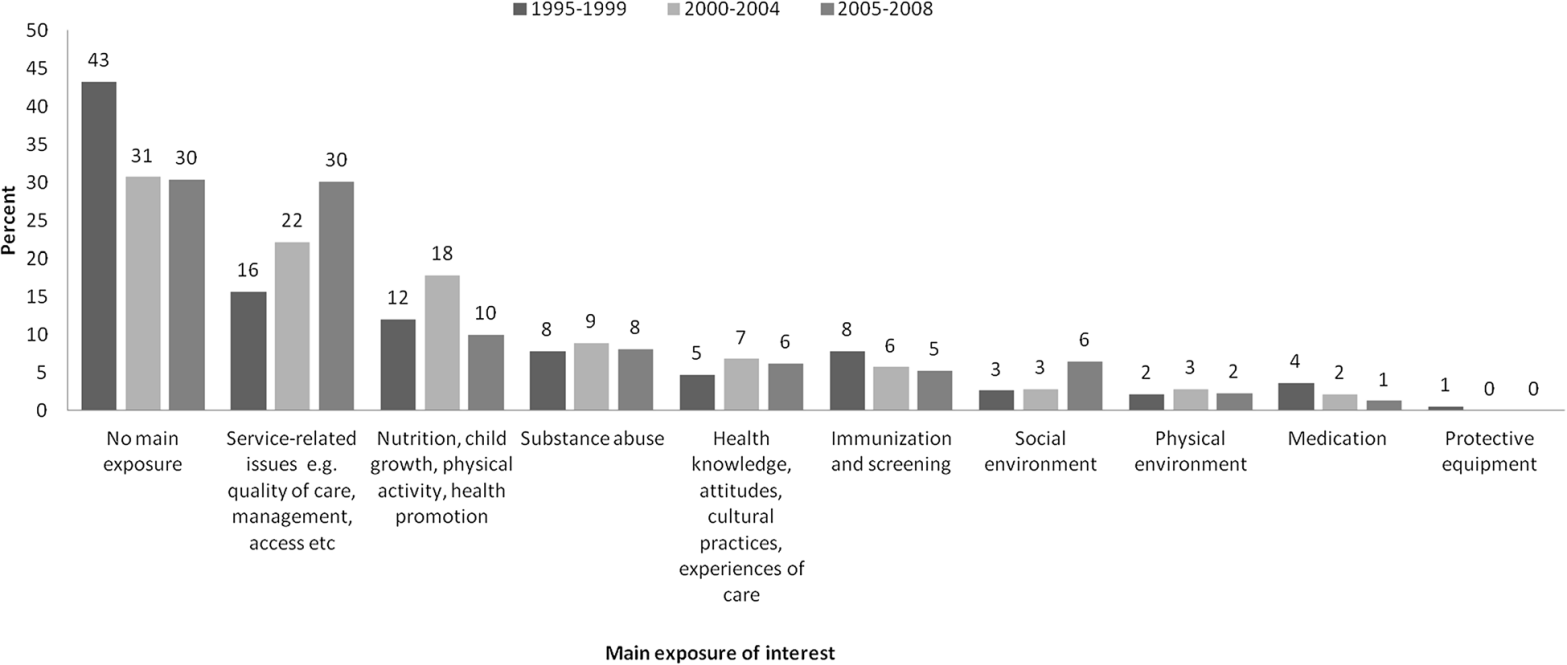
Proportion of publications in each exposure category across each time period.

Figures 3 and 4 describe the proportion of publications in each outcome and exposure category across the three time periods. Between 1995 and 2008, there were increases in the proportion of publications investigating cardiovascular and related diseases (19% to 24%) or mental health (3% to 8%), as well as the proportion of publications classified as having no main outcome of interest (17% to 22%) (Figure 3). There was a decrease in the proportion of publications investigating communicable diseases (21% to 16%), maternal and child health (14% to 5%), as well as those reporting on general service use/mortality data (15% to 7%). For the remaining outcome categories there was no clear change across the three time periods.

Among exposure categories, there was a substantial increase in the proportion of publications assessing service-related issues (16% to 30%) (Figure 4). A small increase was seen in papers assessing the social environment (3% to 6%), whereas there was a drop in publications assessing either immunization and screening (8% to 5%) or medication (4% to 1%). For the remaining exposure categories there was no clear change across the three time periods.

### Network analysis

**Figure 5:**
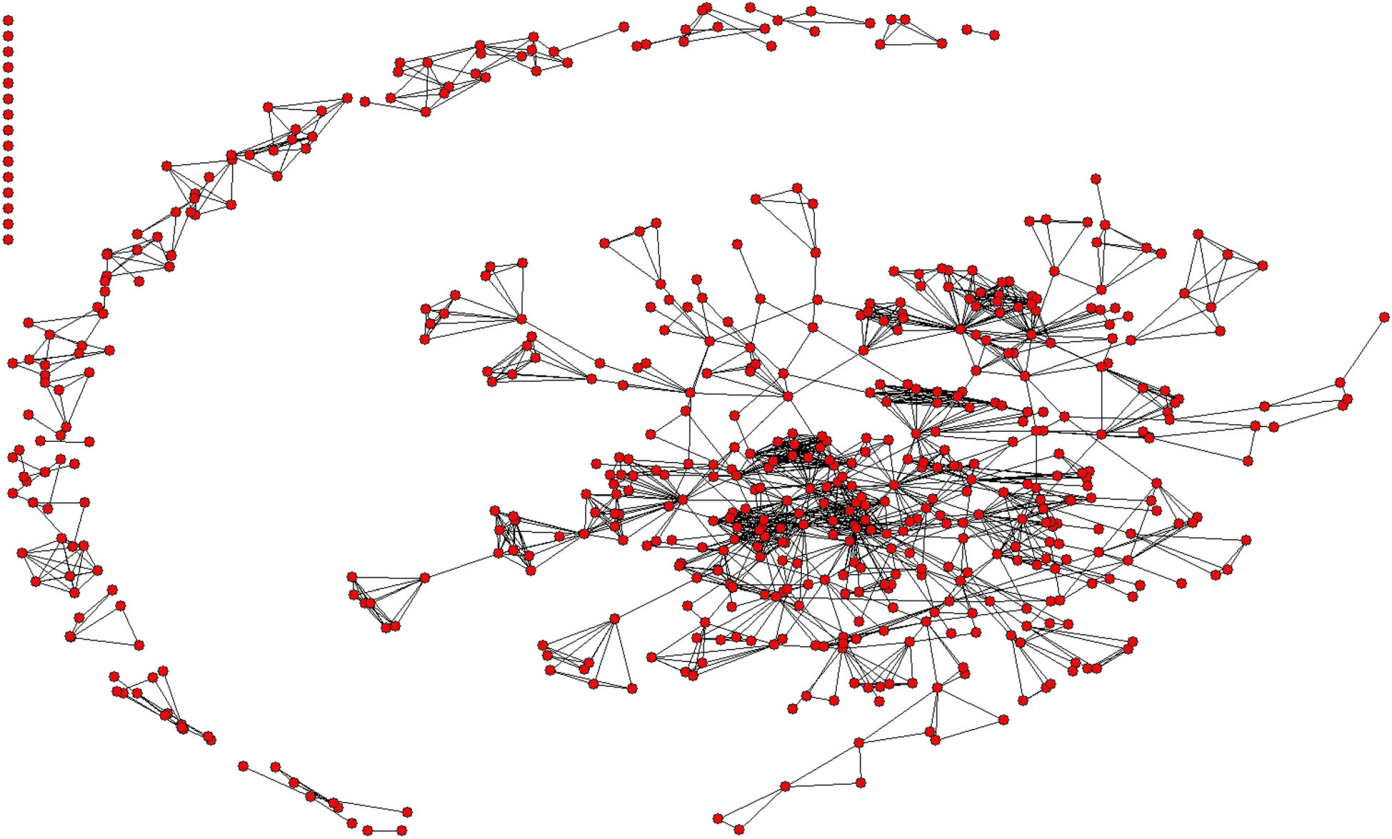
Network map of publications 1995 to 1999.

**Figure 6:**
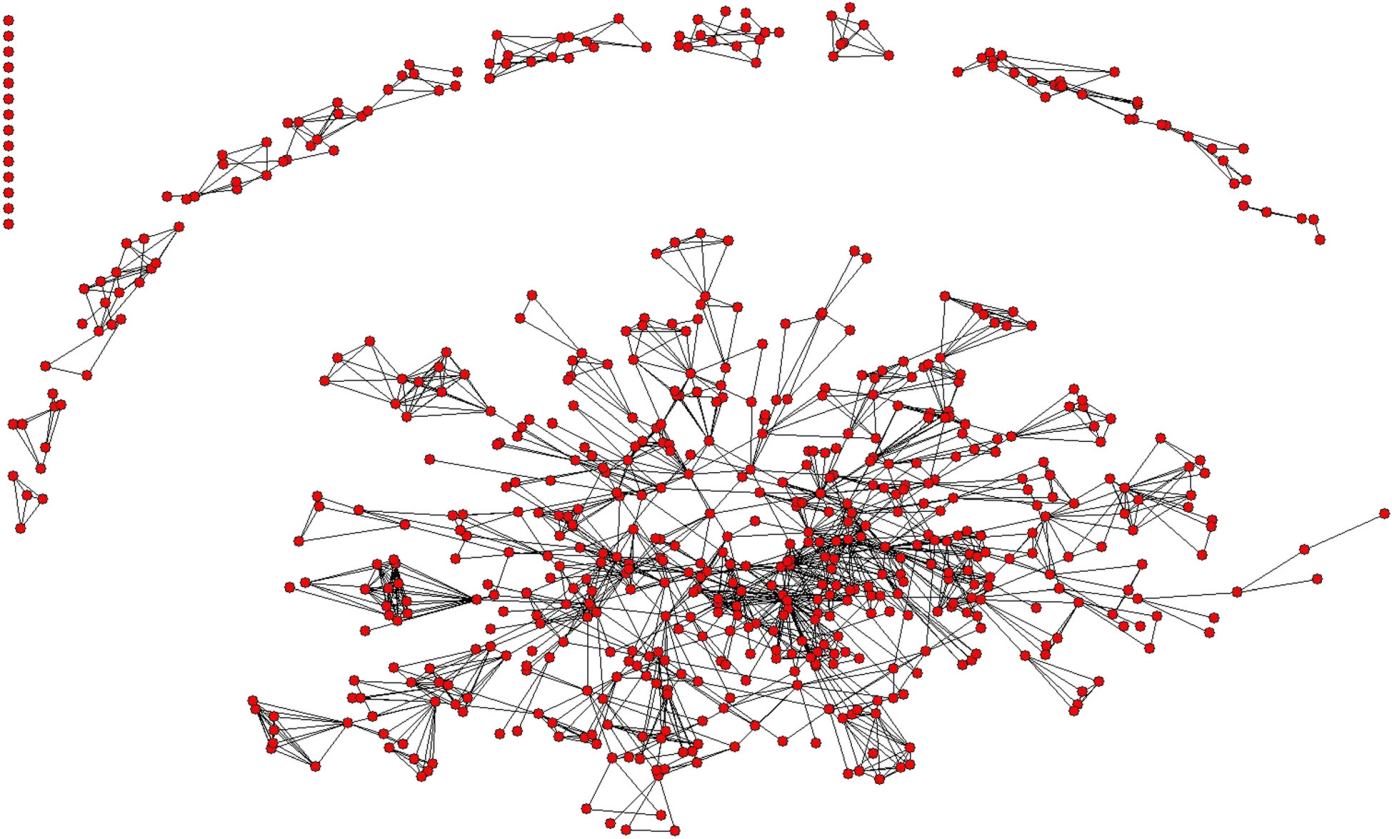
Network map of publications 2000 to 2004.

**Figure 7:**
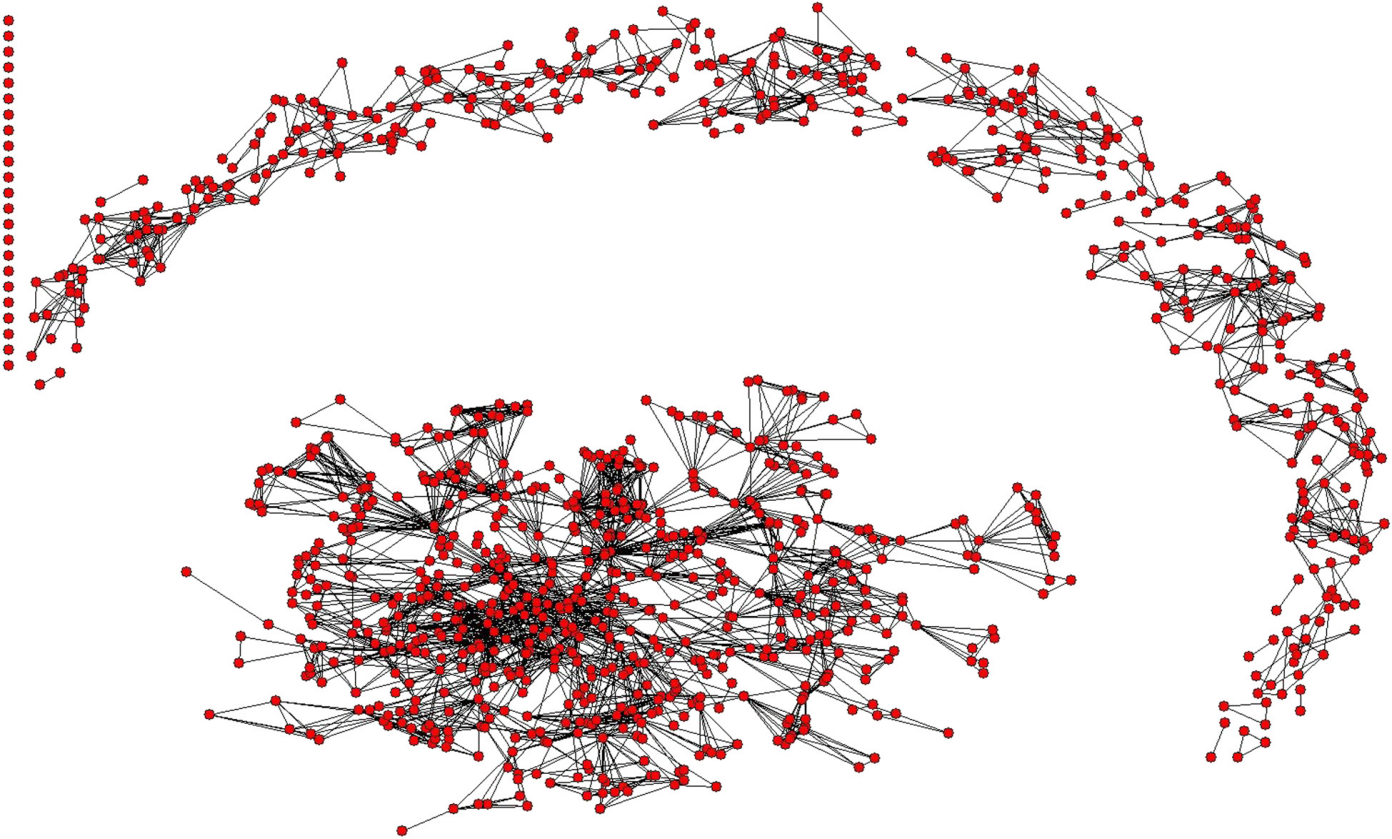
Network map of publications 2005 to 2008.

Figures 5, 6 and 7 show the co-authorship network of eligible publications in the three time periods. Table 2 provides the network component and density measures.

**Table 2:** Network component and density measures for the three time periods

**Measures**	**1995–1999**	**2000–2004**	**2005–2008**
**Whole network**			
Number of authors	583	642	1083
Number of components	57	52	115
Mean (SD) density	0.0053 (0.082)	0.0051 (0.089)	0.0040 (0.084)
**Main component**			
Number (%) of authors in main component	430 (73.8%)	506 (78.8%)	640 (59.1%)
Mean (SD) density	0.0087 (0.105)	0.0075 (0.109)	0.0089 (0.127)
**Other components**			
Number (%) of authors in all other components	153 (26.2%)	136 (21.2%)	443 (40.9%)
Mean number of authors per (non-main) component	2.7	2.7	3.9

SD: Standard deviation.

In the first time period (1995 to 1999), there were 192 publications, with a mean number of authors per publication of 3.7; there were 583 unique authors and 0.33 publications per author. The network (Figure 5) consisted of a core group of 430 individuals, represented by one large cluster (main component) of authors with extensive links with each other. These links were often formed around the work of one highly prolific author. The remaining 153 authors in this time period were located in 56 small components, with a mean of only 2.7 authors per component. The mean density of the overall network was low, and higher but still low for the main component (Table 2); this pattern remained with only small changes across the three time periods.

The subsequent time period, 2000 to 2004 (Figure 6), included 280 publications (mean number of authors per publication 3.8) and 642 unique authors with 0.44 publications per author. The size of the whole network increased slightly regarding the number of authors, but there was a small decrease in the total number of components from 57 to 52. However, the main component grew from 430 to contain 506 authors (Table 2). The mean number of authors in the remaining components was 2.7. This suggests relative stability in the field overall during the periods 1995 to 1999 and 2000 to 2004, with some growth in the development of collaborations around the core group.

In the final time period, 2005 to 2008 (Figure 7), the network became much larger. There were 481 publications (despite the period being only 4 years long) and the mean number of authors per publication was 4.2. The whole network grew to 1,083 authors, about 400 more than in both the earlier time periods, and there were 0.44 publications per author. The number of components also rose to 115 (doubling from the earlier periods). The number of authors in the main component increased to 640, as did the mean size of the other components (3.9 authors) (Table 2). Much more substantial growth in the number of authors, the size of the core group of authors, and the general size of collaborations occurred in this last period compared with the earlier two periods.

The proportion of authors who had only single-author publications in the time period (shown in the upper left hand side of each figure) did not change substantially over the three time periods.

## Discussion

This study has shown the rapid growth of the field of Indigenous health research in Australia in recent decades. The expansion is characterised by an increase in publications and authors, and the size of authorship groups, as well as a shift in the research themes addressed. Social network analyses of authorship patterns also revealed evidence of sustained growth in collaborations over time. Initially, this occurred predominantly around a core group of authors. However, in the last time period, 2005 to 2008, there was a marked increase in the number and size of research groups outside of the core group. Together, these findings indicate that there has been considerable progress in the research sector’s response to building and/or recruiting capacity to address the disproportionate burden of ill-health experienced by Indigenous Australians, although the long-term sustainability of such changes remains to be seen.

Although there have been ongoing policy changes in relation to Indigenous health within the NHMRC (e.g., the recent review and expansion of the *Road Map*[17]), the years 1997 to 2002 have been identified as a period representing accelerated change within the organisation [10]. The catalyst for these changes occurred in 1997 with the establishment of the Aboriginal and Torres Strait Islander Research Agenda Working Group to guide strategic investment in Indigenous health research. Following this, a series of important initiatives were undertaken, including adoption of a set of principles to guide research with Indigenous communities (the “Darwin Criteria”), establishment of the Indigenous health panel in the grant review process, endorsement of the *Road Map*, formal recognition of Indigenous health as a priority area, and policies to support greater representation of Indigenous peoples on NHMRC committees [10]. During this time there were also important initiatives being funded outside of the NHMRC, such as the Primary Health Care Research, Evaluation and Development Strategy, established in 2000 to build the evidence base in Australian primary health care research [18].

The impact of these policy shifts could be direct (e.g., increased funding for specific projects) or indirect (e.g., increased awareness of the importance of Indigenous health research). When assessing the effect on research output, it would be reasonable to expect a lag time of at least a few years, given the often long timelines for completion and dissemination of research. In our study, although we observed a steady increase in publications from 2003, the most rapid growth occurred after 2005. Similarly, the increased size of the research network was most prominent in the last time period examined (i.e., 2005 to 2008). Although it was not possible to determine the number of publications resulting from research directly funded by the NHMRC, these findings suggest that both productivity and partnerships in Indigenous health research may be sensitive to policy changes, including the provision of dedicated and strategic funding.

Among publications identified for this study there was a focus on outcomes related to chronic diseases such as diabetes and cardiovascular disease, and to a lesser extent maternal and child health and communicable diseases. This is not unexpected, as these themes reflect some of the key drivers of the gap in life expectancy between Indigenous and non-Indigenous Australians (e.g., excess chronic disease and infant mortality), as well as the high burden of infectious diseases (e.g., influenza, sexually transmissible infections) experienced by Indigenous peoples [19]. However, several areas appeared to be underrepresented in the current body of research. In particular, there was a dearth of research focussing on mental health, injury, and social and physical environments, despite an excess of ill-health associated with these factors occurring among Indigenous Australians [19,20]. Although the small increase in publications about mental health across the three time periods observed is encouraging, clear gaps in research addressing some of the urgent health needs of Indigenous peoples remain.

Overall, the most common exposure examined among publications was health service-related issues, such as quality of care provided, and there appeared to be a substantial increase in papers assessing these issues across the three time periods. This may have been influenced by the inclusion of health services research as a key theme in the NHMRC’s *Road Map* publication [9] which, for the first time, provided a framework to promote priority-driven research in Indigenous health. The growth in this area is also consistent with a general increase in recent years in funding allocations for health services research in Australia. Between 2000 and 2009 there was a 13-fold increase in NHMRC funding for health services research, compared with smaller increases, in the order of 3- to 5-fold, in other broad research areas including basic science, public health and clinical medicine and science [21]. A corresponding increase in general health services research publications has been observed in Australia in recent years [22] and there has also been increased scholarly output in this field internationally [14].

The network analyses detailed here reveal important insights for researchers, funding agencies and policy makers into the dynamics of the field of Indigenous health research. In each time period, the network had a core-periphery structure, however, the contribution of these components changed over time. The core group, who were highly collaborative and responsible for the majority of outputs, expanded at a steady rate across each time period. The periphery, comprised of small, separate author groups, remained stable in size until the last time period when there was a substantial increase in both the number and size of separate author groups. As a result, the proportion of the research network represented by the periphery increased from 26% in 1995 to 1999 to 41% in 2005 to 2008. Across the three periods there was also an increase in the number of links between authors (both within the core and the periphery), indicating increased collaboration in the field overall.

The observed increase in groups outside of the core group may reflect the growth of new subfields of Indigenous health research such as health services research. These data also suggest that, more recently, researchers who are new to Indigenous health (although not necessarily new to research) have entered into the field without establishing links with the existing core group of Indigenous health researchers. This, along with the fact that nearly two-thirds of authors had only one publication in Indigenous health during the period examined, raises the possibility that these researchers may be less aware of and less likely to participate in the processes of community engagement and long-term relationship building, which are critical elements of working effectively and appropriately in this field. Alternatively, the emergence of individuals in the periphery may have occurred because some members of the core group have not been responsive to the needs of Indigenous communities. While this might not be a new occurrence, the establishment of new research groups (not linked to a researcher with an established track record) may have only been possible in recent years due to greater funding availability.

It is essential to continue to support processes that help ensure the best outcomes are achieved for Indigenous communities, such as appropriately skilled and resourced Human Research Ethics Committees and review of grant applications by an Indigenous panel. There are also innovative funding models, such as that of the Lowitja Institute [23], that facilitate engagement between researchers, Indigenous communities and other key stakeholders. Ongoing institutional support is required to help researchers undertake the appropriate research processes in Indigenous health, a need expressed by researchers in our earlier work [12].

Our finding that most authors had only one eligible publication in Indigenous health in the whole time period is also consistent with our previous work which found that many individuals publishing in Indigenous health research do not consider it to be their primary area of research [12]. This may reflect success in efforts to encourage collaboration in Indigenous health across multiple disciplines or the involvement of researchers with specialist skills in specific projects. It could also reflect an increase in community-led projects, which may require additional time and academic support to develop manuscripts. It is also possible that some authors (particularly junior researchers) deliberately limit their involvement in this field as a career management strategy. This could occur as Australian Indigenous health publications are often poorly cited [22] and less likely to result in traditional measures of research esteem (e.g., international keynote presentations). Finally, it may suggest that the field is not yet developed enough (either in terms of research capacity or sufficient and sustained funding) to support researchers to have Indigenous health as their primary focus of research. Nevertheless, these findings indicate that future strategies to build research capacity in Indigenous health should be targeted across a range of disciplines and types of institutions.

In order to guide future capacity building efforts, further research is needed to determine whether the observed increase in number of collaborations is clustered around certain research institutions and/or key research themes; this was beyond the scope of the current study. Similarly, it is not clear from this study whether the development of the research network has resulted in improved research practices. Previous research examining Indigenous health research proposals among recipients of NHMRC People Support awards (scholarships through to senior research fellowships) found that roughly half of the proposals demonstrated an intent to include Indigenous people in their research, either through consultation processes or as a project partner [10]. Interviews with Indigenous partners and research participants in these and other schemes would be a fruitful area for further research to determine whether current research implementation practices are appropriate. A recent bibliometric analysis of Australian research publications from 1972 and 2008 found a higher rate of growth in Indigenous health-related outputs compared with Australian research overall (average annual percentage increase 15% versus 8%), but there was substantial variability by field, with health services research recording an even higher growth rate (21%) than Indigenous health [22]. Therefore, it would also be valuable to examine co-authorship patterns in other research fields, to determine whether the growth in collaborations seen in Indigenous health reflects adoption of new research models or a general pattern of increased productivity and collaboration in Australian research.

The major strength of our study was the comprehensive searching of a range of electronic databases to capture relevant research publications, including databases which contain sources of grey literature. However, our approach also has several limitations. Analyses were based on identification of individuals who had a research publication in Indigenous health. It is possible that important collaborations were not captured if they did not result in a scholarly output in the time period examined. This approach may have also resulted in fewer Indigenous researchers being identified, as previous research has shown that few Indigenous people are included as authors on journal articles about Indigenous health [24]. In our study, we were unable to determine how many authors identified as Indigenous, and therefore assess the impact of efforts to train more Indigenous researchers. However, recent research suggests that improvements are still needed in this regard, including the development of new models to develop capacity among Indigenous researchers [25]. Further, the network analysis provides an insight into patterns of co-authorship in this field, but is not able to directly answer questions about why this network structure with a large core group and a growing number of smaller groups has arisen, nor to determine the quality of research collaborations.

## Conclusions

The past few decades have witnessed significant changes in funding policy for Australian Indigenous health research. These include increases in overall expenditure as well as a shift towards support for collaborative and priority-driven research. We have shown that this period has coincided with substantial development of the research workforce in Indigenous health, including an increase in research groups, productivity and collaboration between groups. Furthermore, there appears to be a greater focus on some of the identified priority areas for Indigenous peoples, such as health services research, although gaps in other areas still remain. Further efforts to increase research capacity are still required, with a high priority on training Indigenous researchers, as well as a greater focus on evaluation and intervention-based research [17,24]. It will also be important for the NHMRC to continue its commitment to achieving and maintaining a minimum of 5% expenditure on Indigenous health across all funding schemes. Finally, although we have shown rapid expansion of the workforce in Indigenous health research in the past decade, associated with a relatively short period of policy change, sustained commitment to greater support for research in this area is required. This must occur if we are to make real progress in addressing the substantial health disparities between Indigenous and non-Indigenous Australians.

## Abbreviations

CVD: Cardiovascular disease; NHMRC: National Health and Medical Research Council; RAWG: Aboriginal and Torres Strait Islander Research Agenda Working Group; SD: Standard deviation.

## Competing interests

The authors declare that they have no competing interests.

## Authors’ contributions

ARR, JC and BP undertook the literature searches and identification of eligible papers. ARR and JC classified the publications according to research themes. AR prepared the files for the network analyses which were undertaken by JML. All authors participated in the interpretation of findings and drafting of the manuscript. All authors read and approved the final manuscript.
